# Optimized method for fluorine-18 radiolabeling of Affibody molecules using RESCA

**DOI:** 10.1186/s41181-024-00304-9

**Published:** 2024-10-26

**Authors:** Francesco Lechi, Jonas Eriksson, Luke R. Odell, Olivia Wegrzyniak, John Löfblom, Fredrik Y. Frejd, Bo Zhang, Olof Eriksson

**Affiliations:** 1 Department of Medicinal Chemistry, Science for Life Laboratory, Uppsala University, Uppsala, Sweden; 2 PET center, Uppsala University Hospital, Uppsala, Sweden; 3 Division of Protein Engineering, Department of Protein Science, KTH Royal Institute of Technology, Stockholm, Sweden; 4 Department of Immunology, Genetics and Pathology, Uppsala University, Uppsala, Sweden; 5 Affibody AB, Solna, Sweden

**Keywords:** RESCA, Radiochemistry, Aluminium fluoride, PET imaging, Affibody molecule, Peptide

## Abstract

**Background:**

In recent years, the interest in Al[^18^F]F as a labeling agent for Positron Emission Tomography (PET) radiotracers has risen, as it allows for fast and efficient fluorine-18 labeling by harnessing chelation chemistry. The introduction of Restrained Complexing Agent (RESCA) as a chelator has also shown that chelator-based radiolabeling reactions can be performed in mild conditions, making the radiolabeling process attractively more facile than most conventional radiofluorination methods. The aim of the study was to establish optimized conditions for Al[^18^F]F labeling of Affibody molecules using RESCA as a complexing agent, using Z_09591_ and Z_0185_, two Affibody proteins targeting PDGFRβ and TNFα, respectively, as model compounds.

**Results:**

The Al[^18^F]F labeling of RESCA-conjugated Z_09591_ was tested at different temperatures (rt to 60 °C) and with varying reaction times (12 to 60 min), and optimal conditions were then implemented on RESCA-Z_0185_. The optimized synthesis method was: 1.5–2.5 GBq of cyclotron produced fluorine-18 were trapped on a QMA cartridge and eluted with saline solution to react with 12 nmol of AlCl_3_ and form Al[^18^F]F. The respective RESCA-conjugated Affibody molecule (14 nmol) in NaOAc solution was added to the Al[^18^F]F solution and left to react at 60 °C for 12 min. The mixture was purified on a NAP5 size exclusion column and then analyzed by HPLC. The entire process took approximately 35 min, was highly reproducible, indicating the efficiency and reliability of the method. The labeled compounds demonstrated retained biological function for their respective targets after purification.

**Conclusions:**

We present a general and optimized method for Al[^18^F]F labeling of RESCA-conjugated Affibody molecules, which can be widely applied to this class of peptide-based imaging agents. Moreover, radiochemical yields were improved when the labeling was conducted at 37 °C or above. *In vitro* and *in vivo* assessment of the respective tracers was promising, showing retained binding capacity as well as moderate defluorination, which is usually regarded as a potential downside for RESCA-conjugated tracers.

**Graphical abstract:**

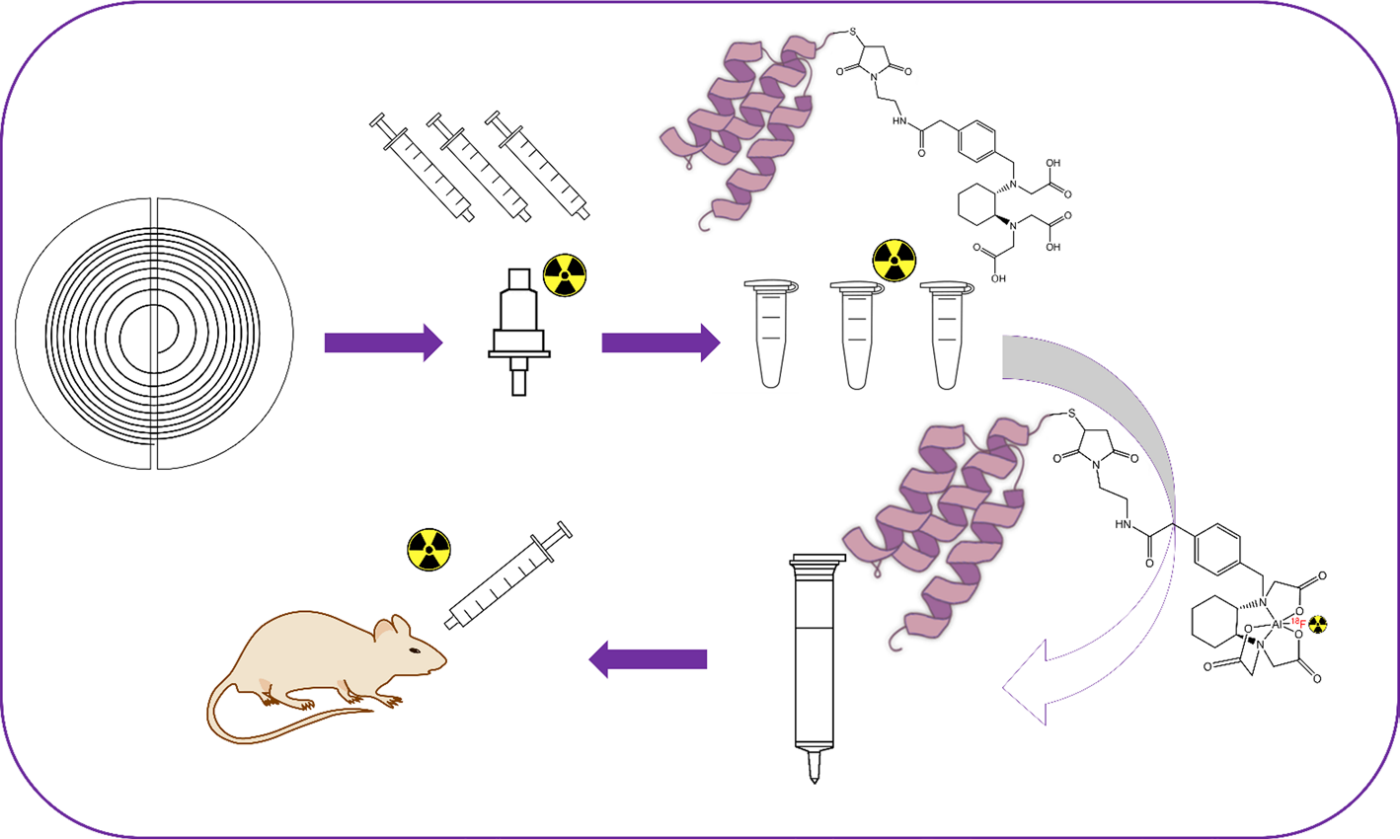

**Supplementary Information:**

The online version contains supplementary material available at 10.1186/s41181-024-00304-9.

## Background

The rising importance of Positron Emission Tomography (PET) as a diagnostic technique in healthcare has brought great interest in method development in radiochemistry and radiolabeling techniques. In the last decade, there has been an increased use of peptide-based scaffolds as imaging agents due to their potential for high affinity combined with a relatively small size and rapid biodistribution. This shift has necessitated the further development of suitable and facile direct and indirect radiolabeling methods [15, 19].

Affibody molecules are a class of peptides, which hold great promise as a general scaffold for PET imaging and radiotherapeutic agents. Affibody molecules are relatively small proteins (58 amino acids, in the range of 6.5–7 kDa) and present a number of favorable traits: a three-helix structure with refolding capability, leading to high thermal stability; high binding affinity in the pM-µM range; fast kinetics and clearance; straightforward functionalization and conjugation with a number of linkers and chelators [11, 17, 18]. Several Affibody molecule-based PET agents are in clinical development, primarily using gallium-68 complexation of 1,4,7,10-tetraazacyclododecane-1,4,7,10-tetraacetic acid (DOTA) or similar macrocyclic chelators. However, gallium-68 has a relatively short half-life of 68 min, making distribution to remote imaging sites challenging, and the high positron energy yields images with non-optimal spatial resolution. Therefore, there is an interest in developing radiolabeling using alternative radionuclides such as fluorine-18 [12, 13].

The Al[^18^F]F-RESCA synthesis method is an efficient approach for chelator-based radiolabeling of peptides and biologics [4]. It allows facile radiolabeling of molecules conjugated with a restrained complexing agent (RESCA) chelator with fluorine-18, an isotope well-suited for PET imaging thanks to its favorable physical half-life (109.7 min) and short positron range, which results in excellent image resolution. The longer half-life, compared to carbon-11 and gallium-68, allows for PET imaging with compounds of slower kinetics, where time for the compound to reach the target, bind and clear from the blood stream is crucial for the imaging. Moreover, fluorine-18 allows for centralized production and subsequent transportation with reasonable conservation of the activity, foregoing the need for in situ production of the radionuclide. The Al[^18^F]F-RESCA synthesis method is distinctive for its mild reaction conditions and high efficiency, enabling rapid, one-step radiolabeling under Good Manufacturing Practices (GMP). Notably, the first peptides labeled with Al[^18^F]F-RESCA have recently entered clinical phase [14].

A major challenge for fluorine-18 labeling of peptides is to find methods that strike a balance between ease of use and efficiency while also preventing degradation of the compound. The introduction of Al[^18^F]F as a labeling agent has proven to be an important step in the direction of faster, one-step labeling strategies; however, using it in combination with conventional chelators such as NOTA, that require temperatures up to 100 °C, prevents applications towards a vast array of heat-sensitive peptides of potential biological interest. In this regard, the Al[^18^F]F-RESCA synthesis method represents an attractive option, as it enables fluorine-18 labeling as low as at room temperature [3–5].

The ability to perform fluorine-18 labeling at room temperature has been one of the major appeals of RESCA as a chelator for peptide-based radiotracers, and there has been limited interest in exploring whether increasing the temperature could give any significant benefits in terms of radiochemical yield (RCY). It has also been shown that the RESCA-Al[^18^F]F chelate resists temperatures up to 50 °C [2, 20]. This represented an interesting gap to investigate, especially since Affibody molecules exhibit variable thermal stability. Thus, a better understanding of the efficiency of the Al[^18^F]F-RESCA synthesis method at a broader temperature range could be used to optimize the radiolabeling conditions for different Affibody molecule based tracers.

**Fig. 1:**
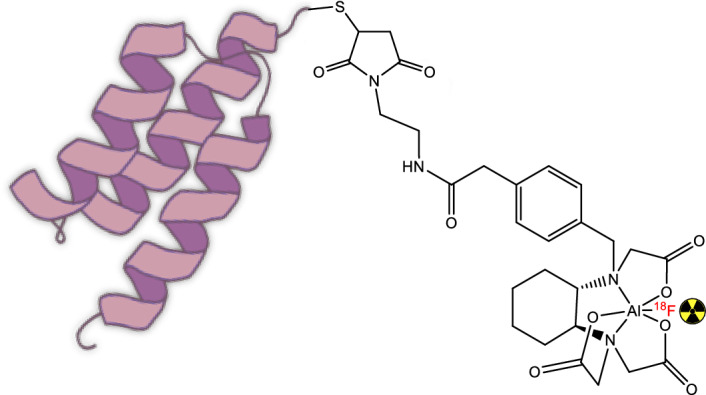
General structure of Al[^18^F]F labeled RESCA-conjugated Affibody molecules

Affibody molecules have been labeled using this strategy in the past [4, 21], making it a suitable avenue for evaluating the chemical and biological properties of Affibody-molecule-based tracers (Fig. 1). The present study explores and optimizes a general protocol for the Al[^18^F]F radiolabeling of RESCA-conjugated Affibody molecules, using two model compounds: Z_09591_, an Affibody molecule targeting platelet derived growth factor receptor β (PDGFRβ) [9, 21], and Z_0185_, which targets the cytokine tumor necrosis factor α (TNFα) [7].

## Material and methods

All reagents and solvents for synthesis and quality control (QC) were purchased from Merck (Germany), Honeywell (North Carolina, USA), Fresenius (Sweden), Thermo Fisher Scientific (Massachusetts, USA). Fluorine-18 was cyclotron produced (Scanditronix, Sweden) by proton bombardment of ^18^O-enriched water (Rotem Industries, Israel). The [^18^F]fluoride transferred to the synthesis hotcell was trapped on a Sep-Pak Accell Plus QMA Plus light cartridge (Waters, Massachusetts, USA). All reactions were performed in Eppendorf Protein LoBind tubes (Eppendorf, Germany). Heating was implemented via heating block on a custom TPS system, while agitation was tested in a VWR Analog Heatblock (VWR, Pennsylvania, USA). Purification was performed via size exclusion chromatography using NAP5 columns (Cytiva, Sweden). Analytical HPLC was performed on an Agilent 1290 Infinity II system (Agilent Technologies, California, USA) using a Vydac 214MS C4 (5 µm, 300 Å, 50 × 4.6 mm) column (Phenomenex, California, USA). Schemes were created with ChemDraw 21.0.0 (PerkinElmer, Massachusetts, USA).

### Synthesis of RESCA-conjugated Affibody molecules

RESCA-conjugated Affibody molecules Z_09591_ and Z_0185_ were synthesized by Almac (UK). The Affibody molecules Z_09591_ and Z_0185_ were synthesized by solid phase peptide synthesis (SPPS) in a minimized format consisting of 58 amino acids (6.5–7 kDa) comprising a three-dimensional structure of three alpha helixes [11]. The amino acid sequence of the Affibody molecule scaffold is highly conserved, except for 13 residues on helixes 1 and 2 which comprise the binding surface and thus are unique for Z_09591_ and Z_0185_, respectively. Helix 3 is not involved in binding but is critical for the stability and refolding properties of the peptide scaffold. Both peptides present a unique cysteine residue at position 59 at the C-terminal, where the RESCA moiety was added (without spacer) using enantiomerically pure (+)-H3RESCA-maleimide (Chematech, France). The product, supplied as a TFA salt, was purified by preparative HPLC, aliquoted in lyophilized form (100 µg/vial) and stored at −80 °C before use. The final products had a purity of >95%, as assessed by HPLC (Supplementary Figure 1, 2). Identity was verified by Electrospray Mass Spectrometry (Supplementary Figure 3, 4).

### ***Production of Al[***^***18***^***F]F***

**Fig. 2:**
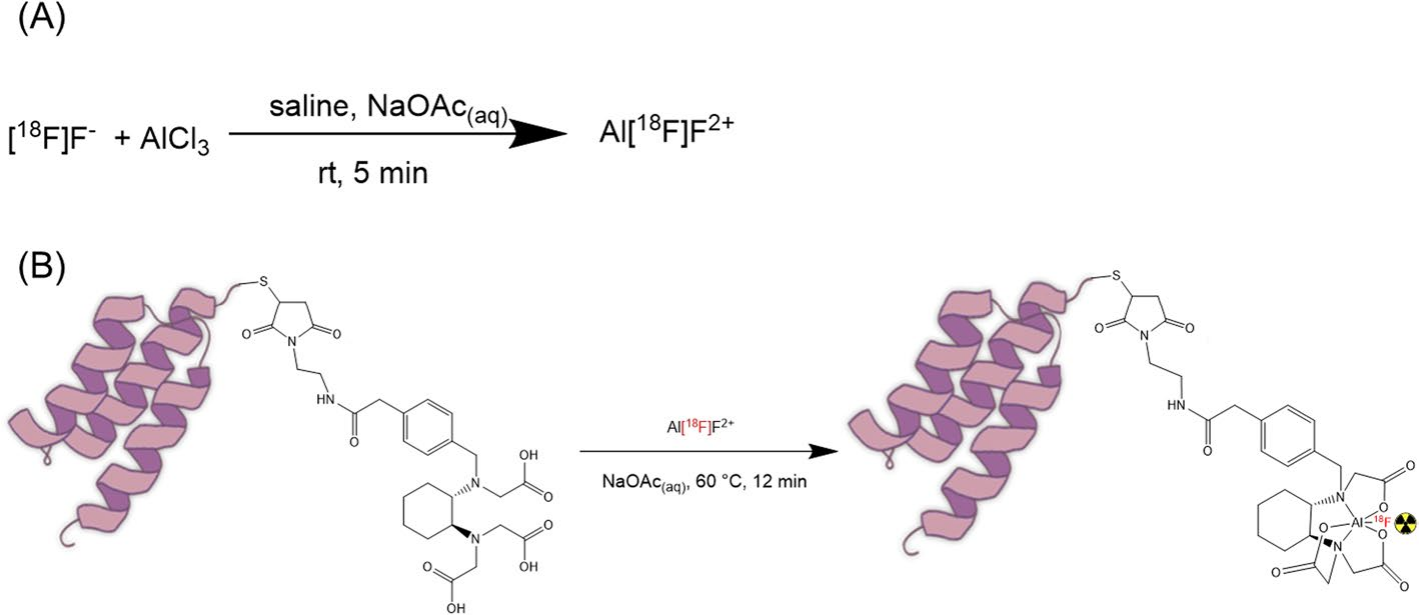
**A** Formation of Al[^18^F]F^2+^. **B** Fluorine-18 labelling of RESCA-conjugated Affibody molecules using optimized reaction conditions

[^18^F]Fluoride was produced by the ^18^O(p,n)^18^F nuclear reaction by proton irradiation of ^18^O-enriched water (17 MeV, Scanditronix MC-17). Approximately 1.5–2.5 GBq of [^18^F]fluoride was trapped on a Sep-Pak Accell Plus QMA Plus light cartridge and then eluted with saline and divided into three fractions (200 µL) in low protein binding vials (1.5 mL Protein LoBind, Eppendorf). The second fraction, containing the highest activity, was added to an aqueous solution of AlCl_3_ (2 mM, 6 μL) in NaOAc (4.65 pH), agitated to ensure thorough mixing and left to react at room temperature (rt) for 5 min (Fig. 2A).

### Optimization of radiolabeling

The RESCA-Z_09591_ Affibody molecule was selected as a model compound for optimizing the labeling protocol. All optimization experiments were performed under metal-free conditions. The effect of temperature and agitation was assessed by performing radiolabeling at room temperature, 37, 50 and 60 °C. Agitation requirements were also investigated at rt. Experiments repeated three times were performed where 200–300 μg of RESCA-Z_09591_ was dissolved in 200–300 μL of aqueous NaOAc and then divided into five aliquots (~40 μL each). A starting activity of 1.6–2.2 GBq [^18^F]fluoride was eluted from a QMA cartridge, reacted with 7 μL of AlCl_3_ solution, and divided equally into the five aliquots (~40 μL each) which was left to react with the RESCA-Z_09591_ aliquots for 12 min at different temperatures. The ratio of reagents, e.g. ^19^F/^18^F and AlCl_3_ may be important for upscaling of the Al[^18^F]F-RESCA synthesis method, but was not further examined in this study.

Lastly, the effect of the labeling reaction time on RCY was assessed at rt and 37 °C. Four timepoints were selected (15, 30, 45 and 60 min) which were performed in triplicates:

[^18^F]fluoride was eluted from a QMA cartridge with 200 μL saline, then 50 μL of the resulting solution (258–805 MBq) were added to a vial containing 6 μL of AlCl_3_ solution and left to react at rt for 5 min. 100 μg of RESCA-Z_09591_ were dissolved in 100 μL of NaOAc and added to the Al[^18^F]^2+^ containing solution and left to react. Radio-HPLC samples were collected at every time point (Column: Vydac 214MS C4 (5 µm, 300 Å, 50 × 4.6 mm, Phenomenex); Solvent A: AMF 50 mM; Solvent B: ACN; Method: 5 to 80% B gradient over 10 min, 4 mL/min). No purification was performed for these series of experiments.

During the optimization tests, formation of Al[^18^F]F was achieved by transferring the fraction of [^18^F]fluoride containing solution with the highest activity to a vial loaded with AlCl_3_ solution. For later labeling reactions, a one-pot approach was taken, instead pre-loading AlCl_3_ solution into the vial wherein the most [^18^F]fluoride activity was expected to be eluted. This approach showed to be beneficial in terms of speed and practicality, and was preferred for all subsequent syntheses.

Through this series of scouting experiments, the optimized protocol described below was established.

### Radiolabeling of RESCA-conjugated molecules

The solution containing Al[^18^F]F (Approx. 1.5–2.5 GBq, 200 μL) was mixed with RESCA-Z_09591_ or RESCA-Z_0185_ (100 μg, 14.09 nmol) in aqueous NaOAc (pH 4.65, 100 μL) and left to react for 12 min (Fig. 2B). The resulting labeled conjugate was then purified by loading the solution onto a NAP5 column size exclusion cartridge and eluted portion-wise with a solution of 10% EtOH in phosphate-buffered saline (PBS) and collected in 400, 600 and 500 μL fractions. The second fraction contained the product with highest radiochemical purity and activity concentration, and was used in the biological evaluation studies. Labeling of the RESCA-functionalized Affibody molecules were performed under metal-free conditions.

Radiochemical yield (RCY) was calculated by integration of radio-HPLC peaks of samples taken before purification. The HPLC column used was Vydac 214MS C4 (5 µm, 300 Å, 50 × 4.6 mm, Phenomenex), eluted with a gradient of 5% to 80% acetonitrile in water/0.1% TFA over 10 min, at a flowrate of 4 mL/min. The retention times were 4.0 min for Al[^18^F]F-RESCA-Z_09591_ and 4.2 min for Al[^18^F]F-RESCA-Z_0185_.

The isolated decay-corrected radiochemical yield (RCY_isolated_) was estimated as follows: (*A*_*(second fraction)/*_*A*_*(total)*_*) x 100*), where A_(total)_ was the sum of the measured activity values of the three purified fractions and the NAP5 column after purification.

Samples were taken before and after purification and analyzed by radio-HPLC, to monitor the reaction in the crude reaction mixture and to assess the radiochemical purity (RCP) of the product solution.

The pH was monitored via pH paper strips. The Al[^18^F]F solution had a pH of 5.5, while both the reaction mixture and the purified product had a pH of 4.5, aligning with the method reported in previous literature.

Total synthesis time following [^18^F]fluoride elution from the QMA cartridge was approximately 35 min.

### ***HPLC recovery of ***^***18***^***F***

To ensure the accuracy of the radiochemical yield assessment for Al[^18^F]F-RESCA-Z09591 performed by radio-HPLC and to confirm that no activity remained on the HPLC column, column recovery was determined. The evaluation was conducted under two conditions: once with the HPLC column in place and once without it. During the analysis, the eluted mobile phase was collected and subsequently measured using a sensitive gamma well counter after 12 to 14 hours. The column recovery, performed twice, was found to be 97.1% ± 0.5%.

### In vitro studies of RESCA-conjugated Affibody molecules

#### ***In vitro autoradiography binding studies using Al[***^***18***^***F]F-RESCA-Z***_***09591***_

The biological activity of Al[^18^F]F-RESCA-Z_09591_ was tested to ensure preserved binding functionality. *In vitro* autoradiography was conducted on frozen sections from either U87 cell pellets, a cell line with known expression of PDGFRß, or previously acquired tissue biopsies (liver, spleen and muscle) from mice. These mice were either healthy or treated for 6 weeks with intraperitoneal (i.p.) carbon tetrachloride (CCl_4_) to induce a model of liver fibrosis accompanied by overexpression of PDGFRß on activated hepatic stellate cells. Separate biopsies from the mice were fixed in formalin, embedded, sectioned and stained for collagen (Sirius red, masons trichome (MTC)) as well as PDGFRß to confirm disease development and target receptor expression, as described in detail previously [21].

Briefly, 20 µm frozen sections were thawed to room temperature, and allowed to equilibrate in incubation buffer (PBS + 1% bovine serum albumin (BSA), 150 mL) for 10 min. This was performed either in buffer only, or in buffer supplemented with Cys-Z_09591_ (1 µM) to saturate the PDGFRΒ on the sections. Subsequently, Al[^18^F]F-RESCA-Z_09591_ (5 nM, approximately 1 MBq/mL) was added and allowed to bind to the section for 60 min. Post-incubation, the sections were washed twice for 1 min in PBS + 1% BSA buffer without added radioactivity. An additional wash was performed for 1 min in PBS, followed by dipping the sections in Milli-Q water. The sections were then dried in a heating cabinet at 37 °C for 15 min, and then exposed overnight to a digital phosphorimager plate (BAS-MS, Fuji-film) alongside radioactive standards prepared from the incubation buffer to allow quantification of the resulting autoradiograms. The plates were scanned and digitalized using a Phosphor Imager (Amersham Typhoon FLA 9500 Phospor Imager, GE). The autoradiograms visualized and analysed using ImageJ (National Institutes of Health, US).

#### ***In vitro plasma stability of Al[***^***18***^***F]F-RESCA-Z***_***09591***_

Leakage of Fluorine-18 from the RESCA chelator has been reported and may cause unwanted image artefacts such as non-specific uptake in bone tissue. Furthermore, it is not known if the stability of the enantiomerically pure (+)-H3RESCA used here is different from the racemic mixture used in most of the literature. The stability of Al[^18^F]F-RESCA-Z_09591_ in rat or human plasma samples was therefore evaluated.

Briefly, rat plasma (acquired by heart puncture from healthy Sprague Dawley rat) or human plasma (anonymous samples from the local blood bank) was mixed with Al[^18^F]F-RESCA-Z_09591_ (in a 9:1 volume ratio). The mixture was incubated at 37 °C for 0, 5, 10, 30, 60, 90 or 120 min. Next, the samples were placed on ice and the plasma proteins were precipitated by adding acetonitrile in an 1:1 ratio. The supernatant was filtered through a low protein binding 2X0.2 µm nylon membrane and the resulting solution analyzed by HPLC (5-10µl solution injected). The details of the plasma stability assays and the HPLC protocol was described in detail previously [21].

#### ***In vitro ELISA binding studies using Al[***^***18***^***F]F-RESCA-Z***_***0185***_

A Copper Coated High-Capacity Plate was coated with 10 μg/ml his-tagged TNFα by incubating for 1 hour at room temperature, then washed 3 times with 200 μL PBS, 0.05% Tween®-20 Detergent. Blocking non-specific sites was achieved by incubating with 100 µL PBS 1% BSA for 15 min and followed by 3 times washes. Al[^18^F]F-RESCA-Z_1085_ was delivered, was added to each well (100 µL of tracer solution, 500 kBq) and incubated for 60 min at room temperature. After washing 3 times with 200 μL PBS, 0.05% Tween®-20 Detergent, all wells were detached, and the radioactivity was measured by the well counter. PBS was used as the negative control for incubation instead of his-TNF, and DiGeorge Syndrome Critical Region Gene 2 (DGCR2) was used as the random protein for confirming the specificity of the tracer. To investigate the competitive binding with a known TNF inhibitor, etanercept was added same time as the tracer at a concentration of 80 nM.

### ***In vivo studies of RESCA-conjugated Al[***^***18***^***F]F-RESCA-Z***_***0185***_

#### Animal handling and housing

Mice (Balb/c, n=10, female, 24.4±1.1g) were housed at five per cage lined with GLP Aspen Bedding (Tapvei, Estonia) in individually ventilated cages with free access to water and food*.* Animals were housed under a constant temperature of 22 °C and humidity (50%) in a 12 h/12 h light/dark cycle. The Animal Ethics Committee of the Swedish Animal Welfare Agency approved all experimental protocols. The procedures were performed in agreement with the ARRIVE (Animal Research: Reporting of *In Vivo* Experiments) recommendations and institutional guidelines (“Uppsala University guidelines on animal experimentation”, UFV 2007/724).

#### PET scanning and ex vivo biodistribution studies

Mice were administered a target bolus dose of 1 MBq of Al[^18^F]F-RESCA-Z_0185_ in the tail vein (1.0±0.2 MBq, corresponding to approximately 1 µg peptide). All mice were euthanized 60 min after injection for *ex vivo* assessment of tracer biodistribution. The mice were dissected, and their organs (blood, lungs, liver, spleen, kidneys, muscle, bone (femur), gastrointestinal tract, remaining body, tail) were collected, weighed and radioactivity content assessed by a gamma counter (inhouse design, Uppsala Imanet). Bone was of special interest, as Al[^18^F]F-RESCA labeled compounds has been posited to potentially “leak” fluorine-18, which then could accumulate in bone tissue by mimicking hydroxide ions in hydroxyapatite.

The uptake in individual tissues was converted to Standardized Uptake Values (SUV) by correcting for animal weight and injected dose, to enable comparison between individuals.

Two of the mice were scanned by PET/CT postmortem, before organ distribution. This design was used to give PET images, while still being able to have comparable ex vivo gamma counter data as the rest of the animals. The mice (n=2) were placed with the entire body in the field of view of a nanoPET/MRI scanner (Mediso, Hungary) and scanned for 20 min. Next, the movable bed was transferred to a nanoSPECT/CT scanner (Mediso, Hungary), and a 5 min CT scan was performed. SUV corrected PET/CT images were visualized using the Carimas software (Turku PET Center).

#### Statistical Analysis

The results are presented as mean ± standard deviation (SD). GraphPad Prism (version 10.1.0, GraphPad Software, San Diego, CA, USA) was used to conduct statistical analyses. Differences between groups were performed using Students T-test or one-way ANOVA (*p*<0.05).

## Results

### Optimization of radiochemistry

**Fig. 3:**
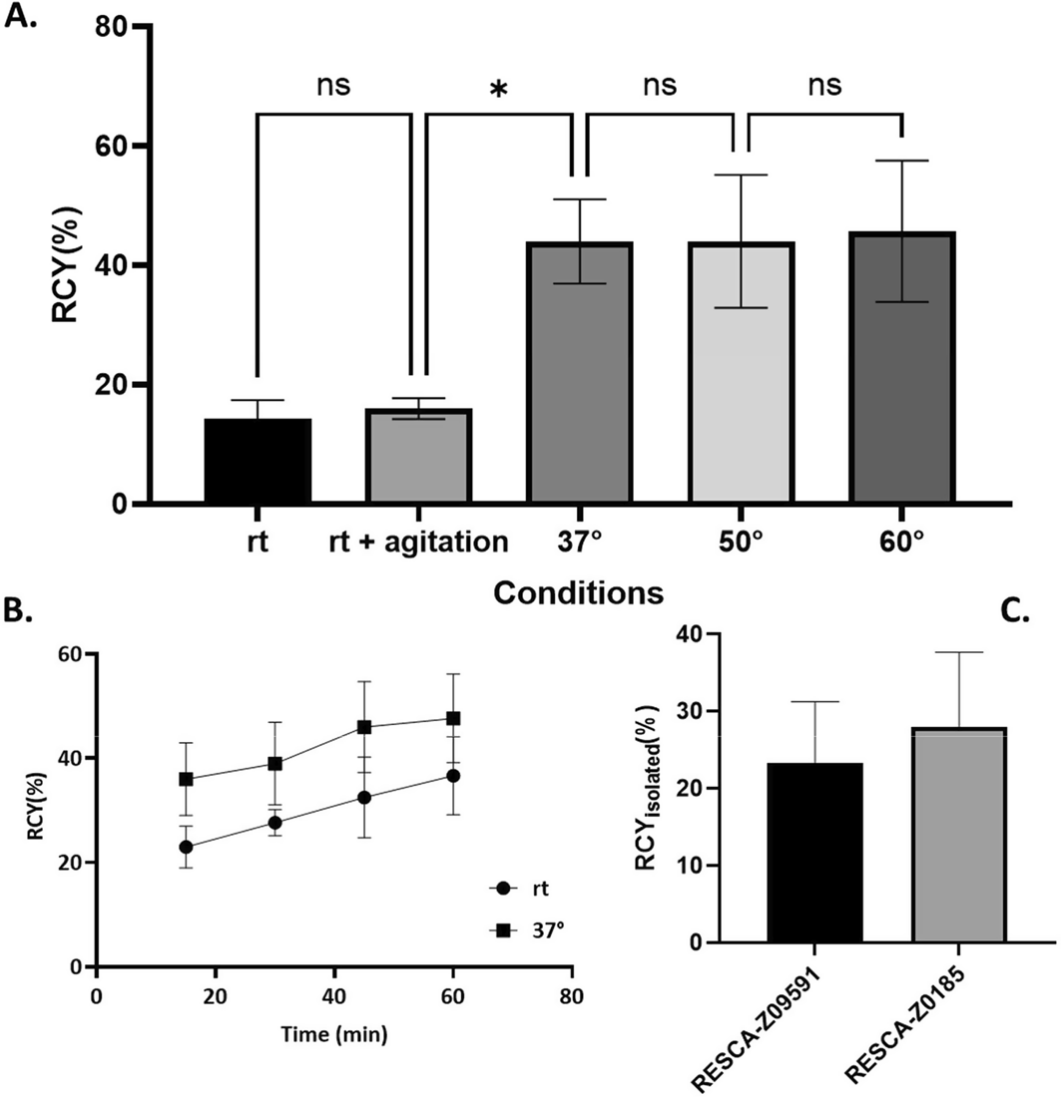
**A** Results of the comparative reaction temperature performed for Affibody molecule RESCA-Z_09591_. Every condition was repeated three times. The starting activity was 1.6–2.2 GBq for each series. An increase in RCY was noticeable when increasing temperature from room temperature to 37 °C (* indicates *p*<0.05 as assessed by one way ANOVA). **B** Results of labeling reaction time assessment. N=4 timepoints: 15, 30, 45, 60 min; reaction tested at rt and 37 °C (N=3 repeats each). Increasing reaction time at rt appears to yield no benefits in terms of RCY that couldn’t be already achieved with mild heating. **C** A comparison between decay-corrected RCY yields for the two tracers in similar conditions (n=6 for RESCA-Z_09591_ and n=4 for RESCA-Z_0185_).

Initially, a series scouting experiments were performed to establish optimal synthesis parameters. The first step involved investigating the effects of temperature (rt, 37 °C, 50 °C and 60 °C) and agitation on the RCY. Subsequent tests assessed the impact of varying the starting activity over concentration of tracer ratio. Notably, a significant increase in RCY was observed when the reaction temperature was increased from room temperature to 37 °C; however further increases had minimal impact on the RCY (Fig. 3A). Agitation at room temperature was found to yield no significant benefits. The significance of the difference in RCY under various conditions was determined using an ordinary one-way ANOVA.

Further experiments were conducted to investigate the effects of extended reaction time on the RCY. The aim was to determine whether or not increasing the reaction time could be an alternative strategy to heating. This was achieved by performing the labelling of the RESCA-Z_09591_ Affibody molecule three times at room temperature and at 37 °C and collecting radio-HPLC samples at the 15 min, 30 min, 45 min and 60 min time points. Purification was not performed for this assay, as it was reasoned that changing the time parameter would not affect it. Results showed that, while a longer reaction time improved RCY in the long run, even after 60 min of reaction at room temperature the resulting increase RCY was in the same order of magnitude of that which was obtained by heating at 37 °C over 15 min. In other words, total synthesis time at room temperature would need to be tripled in order to attain the same RCYs that were achieved with mild heating (Fig. 3B).

The parameters established in the various tests performed on RESCA-Z_09591_ were then applied to the preparation of a second Affibody molecule Al[^18^F]F-RESCA-Z_0185_. These conditions afforded a comparable yield of both ^18^F-labeled compounds (Fig. 3C).

When performing syntheses to be used for in vitro and in vivo assays, size-exclusion purification via NAP5 columns proved to be reliable and practical: values of RCP>99% were attained consistently for both radiotracers.

Apparent molar activity (A_m_) was in accordance with previously published studies [3], and was in the range of 13.6–56.6 GBq/µmol when using a starting amount of 14 nmol of RESCA-conjugated Affibody molecule.

### ***Biological activity of Al[***^***18***^***F]F-RESCA-Z***_***09591***_

**Fig. 4:**
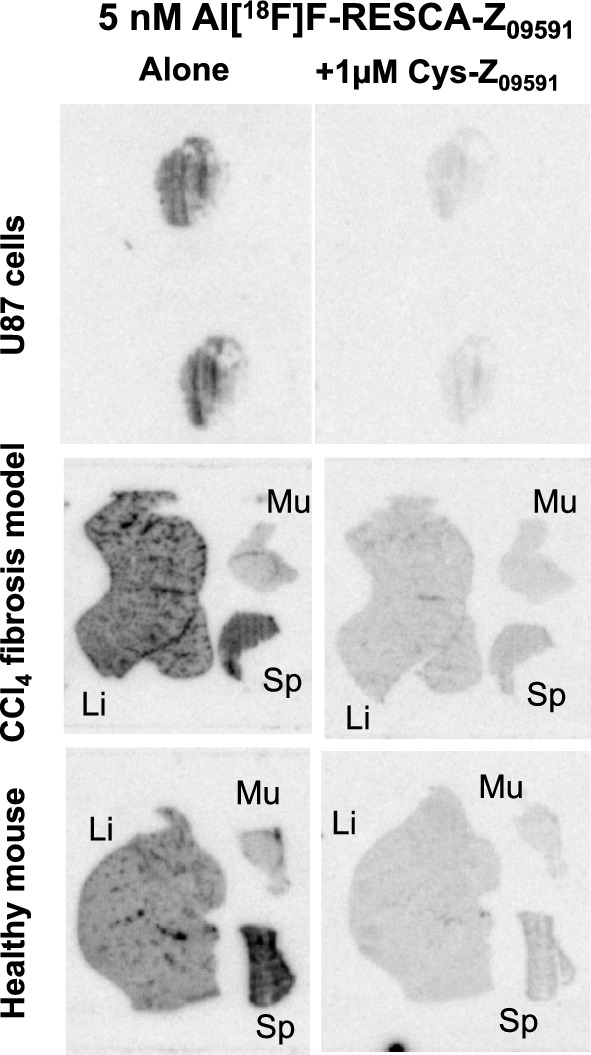
Autoradiography binding studies using Al[^18^F]F-RESCA-Z_09591_. Al[^18^F]F-RESCA-Z_09591_ bound to PDGFRß positive U87 cells in a manner reversed by co-incubation with unlabeled Cys-Z_09591_ in excess (top panels). Similarly, Al[^18^F]F-RESCA-Z_09591_ could detect fibrogenic lesions with increased expression of PDGFRß in liver from mice treated by CCl_4_ (middle row), compared with healthy mice (bottom row). Mouse spleen, positive for PDGFRß also demonstrated increased tracer binding by a mechanism blockable by Cys-Z_09591_

Al[^18^F]F-RESCA-Z_09591_ retained its binding interaction towards PDGFRß following radiolabeling with Al[^18^F]F. This was demonstrated in both PDGFRß-expressing U87 cells and a model of liver fibrosis (Fig. 4). Al[^18^F]F-RESCA-Z_09591_ retained binding to U87 cell sections (Fig. 4, top panels), which could be abolished by co-incubation with excess Cys-Z_09591_. The compound also demonstrated increased binding to fibrotic mouse liver tissue (Fig. 4, middle panels), compared to healthy liver tissue (Fig. 4, bottom panels). Again, this binding was inhibited by pre-blocking of the PDGFRß. Notably, the binding was localized to the hepatic sinusoid capillaries, where both collagen deposition and PDGFRß expression were confirmed through histology and immunohistochemistry (Supplementary Figure 5).

### ***Plasma stability of Al[***^***18***^***F]F-RESCA-Z***_***09591***_

Al[^18^F]F-RESCA-Z_09591_ demonstrated high stability in vitro in both rat and human plasma for up to 120 min, with minimal degradation or leakage of Fluorine-18 (Supplementary Figure 6).

### ***Biological activity of Al[***^***18***^***F]F-RESCA-Z***_***0185***_

RESCA conjugation of Z_0185_ did not interfere with its interaction towards recombinant human TNF, as indicated by an Surface Plasma Resonance (SPR) assay (Supplementary Methods and Supplementary Figure 7A). RESCA-Z_0185_ exhibited an affinity in the single digit nanomolar range, but only modestly inhibited the binding between etanercept and TNF (Supplementary Figure 7B).

Al[^18^F]F-RESCA-Z_1085_ also retained interaction towards recombinant human TNF after radiolabeling, using an ELISA style assay. The binding to wells containing anchored TNF was strongly increased compared to wells containing no (background) or a random protein (Supplementary Figure 7C). Addition of etanercept partly inhibited the interaction of Al[^18^F]F-RESCA-Z_1085_ towards TNF, in line with the results from the SPR assay.

**Fig. 5:**
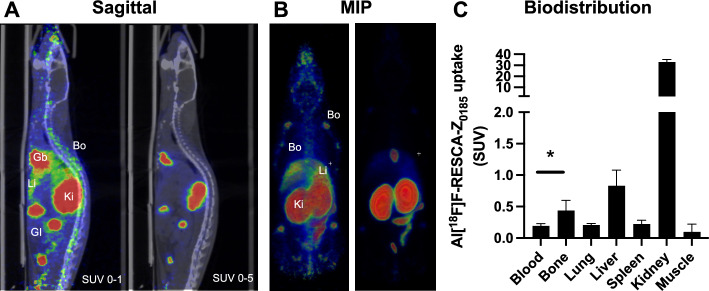
*In vivo* biodistribution of Al[^18^F]F-RESCA-Z_0185_ in mouse. Biodistribution of Al[^18^F]F-RESCA-Z_0185_ as evaluated in mice by PET scanning and ex vivo organ distribution, demonstrated rapid renal clearance and low background in most tissues (**A**–**B**). There was a small increase in uptake in bone tissue, indicating a degree of defluorination (**C**). MIP = Maximum Intensity Projection. A star (*) indicates *p*<0.05 as assessed by the Student’s T-test)

*In vivo* biodistribution of Al[^18^F]F-RESCA-Z_0185_ was evaluated in mice, 60 min after injection, by both PET scanning and *ex vivo* organ gamma counter measurement. Al[^18^F]F-RESCA-Z_0185_ exhibited rapid clearance from blood and most tissues, combined with almost exclusively renal excretion (Fig. 5A-C). Strong accumulation was seen in the renal cortex, likely indicating trapping of charged Al[^18^F]F. Binding in bone tissue was also observed, with an SUV of approximately 0.5 (approximately 2 times the tracer concentration in blood), indicating a degree of defluorination and binding to apatite (Fig. 5A-C). This was seen both in PET images (e.g. femur, humerus, spine), and the gamma counter measurements (femur).

## Discussion

As fluorine-18 suggests excellent theoretical improved performance as a PET radionuclide compared to e.g. gallium-68, exploring the potential of the Al[^18^F]F radiolabeling method of peptides and proteins is of high interest. This method efficiently combines the simplicity of chelation chemistry with the high imaging quality offered by fluorine-18 [3]. Additionally, cyclotron produced fluorine-18 can offer significantly higher activity than germanium-68/gallium-68 generators, suggesting a potential to scaling up production when necessary [14]. However, this does not diminish the utility and relevance of gallium-68, which remains the most widely used chelating PET-radionuclide in both clinical and research PET applications.

Several procedures for radiofluorination of peptides have been described and extensively investigated, such as indirect labeling by prosthetic groups (e.g. N-succinimidyl-4-[^18^F]fluorobenzoate ([^18^F]SFB) and chelators capable of coordinating fluorine-18 metal complexes (e.g. NOTA). However, each have distinct challenges, such as the need for multi-step reactions and thus increased synthesis time, or conditions that are less than ideal for sensitive biological molecules (high temperatures, organic solvents).

The RESCA chelator was developed to mitigate the challenges outlined above with a focus on allowing one-pot radiofluorination at low temperatures [4]. RESCA enables the coordination of Al[^18^F]F at ambient room temperature, although RCY and complex stability may not be optimized.

Recently, there has been increased application of RESCA in efforts for radiofluorination of PET tracers based on the Affibody molecule scaffold [10, 21]. Affibody molecule imaging tracers have often used traditional labeling methods such as gallium-68 or indium-111, especially in clinical development phase [1, 16]. However, as outlined above, reliable fluorine-18 labeling techniques may be very beneficial for further preclinical and clinical development of imaging agents based on current or future Affibody molecule binders.

Here, we present a general and rapid protocol for radiofluorination of RESCA-conjugated Affibody molecules. Al[^18^F]F coordination of RESCA-conjugated Affibody molecules was successfully achieved at temperatures ranging from room temperature to 60 °C, irrespective of agitation, demonstrating reliability and flexibility towards implementation on different automated synthesis platforms. Additionally, the rapid and efficient purification achieved by the size exclusion NAP5 column consistently gave product solutions with high RCP. The improved RCYs at 37 °C compared to room temperature are particularly noteworthy. This suggests that more heat-sensitive Affibody molecules (e.g. those with melting temperature in the 40–50 °C range) can be successfully labeled using this approach with improved RCYs. In the process of selecting for novel Affibody molecule binders by phage display techniques, it is common to identify many suitable high affinity variants, which may be discarded due to having thermal stability judged as insufficient (such as a melting temperature of <55 °C). This is of course related to finding a lead compound which can tolerate the conditions required for further development e.g. as a PET tracer, and the available radiolabeling methods. Consequently, this new protocol opens up possibilities for re-evaluating high-affinity Affibody molecules that might otherwise be discarded due to insufficient thermal stability when labeled at high reaction temperatures, which have typically been required for fluorine-18 and gallium-68 incorporation.

Increasing the chelation reaction time showed somewhat beneficial, with RCY improving greatly after 60 min. However, this increase comes at the price of losing a high amount of activity to attain RCY values that do not exceed what was already measured when performing the chelation reaction at 37 °C over 12 min. In the context of radiofluorination chemistry, this is highly undesirable. A major strength of the Al[^18^F]F-RESCA method is its simplicity and ease of use. Labeling of RESCA-conjugated Affibody molecules was consistently straightforward and robust [3, 21]. The entire synthesis could be performed using a one-pot approach, further enhancing its practicality and efficiency. This simplicity also renders it a promising candidate for automation, which could potentially improve RCYs and consistency across syntheses, while simultaneously reducing the operator’s radiation exposure compared to manual labeling [6, 8], with the caveat that size-exclusion purification might still require to be performed separately. Nevertheless, a semi-automated approach would still prove beneficial.

Finally, the conjugation of RESCA at the C-terminal of the precursor Affibody molecule did not impair the interaction with the target for either Z_0185_ or Z_09591_, according to SPR [21]. The Al[^18^F]F-RESCA labeled Affibody molecules retained biological function, both *in vitro* and *in vivo*. The *in vivo* performance of Al[^18^F]F-RESCA-Z_09591_ as an imaging biomarker of active liver fibrosis was recently demonstrated separately [21], illustrating how the currently described radiofluorination technique can be applied in a biologically relevant disease model. Initial studies on the biodistribution of TNF-targeting Al[^18^F]F-RESCA-Z_0185_ in mice demonstrated increased but limited uptake in bone tissue (SUV≈0.5 after 60 min, around twice the tracer concentration in blood), indicating moderate levels of defluorination of the tracer. A similar in vivo uptake in mice was previously seen also with Al[^18^F]F-RESCA-Z_09591_ [21], indicating that these observations potentially can be generalized to other Affibody molecules radiofluorinated using the enantiomerically pure (+)-H3RESCA chelator.

Insufficient stability of the Al[^18^F]F-RESCA complex has previously been suggested as a limitation of the technique, especially based on observation in mice and rats, but less so in pigs [21], non-human primates and humans [10]. Furthermore, Al[^18^F]F-RESCA-Z_09591_ here demonstrated excellent stability in human and rat plasma in vitro for up to several hours. Thus, the defluorination of the Al[^18^F]F-RESCA complex is likely dependent on in vivo processes. The biodistribution and defluorination of Affibody molecules radiofluorinated with the optimized Al[^18^F]F-RESCA protocol described here require further investigation.

## Conclusion

In summary, the Al[^18^F]F-RESCA method was successfully employed to radiolabel two different model Affibody molecules, Z_09591_ and Z_0185_. We present a general, facile and optimized fluorine-18 labeling protocol that can be widely applied for RESCA-conjugated Affibody molecules, and fine-tuned depending on the thermal stability of the individual binder. The improvement in RCY observed at temperatures as low as 37 °C suggests that even minor adjustments to the reaction temperature can lead to significant benefits without sacrificing practicality or reaction times.

## Supplementary Information

Additional file1

## Data Availability

The datasets used and/or analysed during the current study are available from the corresponding author on reasonable request.
