# Supplementary material for: Optimized method for fluorine-18 radiolabeling of Affibody molecules using RESCA
Source: EJNMMI Radiopharm Chem. 2024 Oct 26;9:73. doi: 10.1186/s41181-024-00304-9 (PMC11512968; doi:10.1186/s41181-024-00304-9)
Supplement: Supplementary file 1 — Additional file1 [file 41181_2024_304_MOESM1_ESM.docx]

**Optimized method for Flourine-18 radiolabeling of Affibody** **molecules using RESCA**

Francesco Lechi, Jonas Eriksson, Luke R. Odell, Olivia Wegrzyniak, John Löfblom, Fredrik Y Frejd, Bo Zhang, Olof Eriksson

**SUPPLEMENTARY METHODS**

**Analytical Methods for RESCA-conjugated Affibody molecule precursors**

Analytical RP-HPLC was performed on a Waters Alliance system. Column: Luna C18(2) 250 x 4.6 mm id, 5μm, 100Å. Mobile Phase A: Purified Water + 0.1% TFA. Mobile Phase B: Acetonitrile + 0.1% TFA

| Gradient: **Time (min)** | **% A** | **% B** |
| --- | --- | --- |
| 0.00 | 98 | 2 |
| 4.00 | 98 | 2 |
| 38.00 | 10 | 90 |
| 40.00 | 10 | 90 |
| 42.00 | 98 | 2 |

Injection Volume: 5-20 μL. Column Temperature: 30 ºC. Flow Rate: 1.0 mL/min

Wavelength: 214 nm. Sample Temperature: Ambient.

Electrospray mass spectrometry analysis was performed using a SciEx X500B QTOF electrospray mass spectrometer in positive ion mode. Eluent: Purified Water/Acetonitrile (1:1) + 0.1% formic acid. Flow rate: 0.6 mL/min

**Surface Plasmon Resonance (SPR) using *RESCA-Z_0185_***

A Biacore® T200 instrument (Cytiva) was used to evaluate the interactions between the RESCA-conjugated TNFα binding affibody (_Z0185_) and TNFα, to evaluate if the addition of RESCA would impair the biological activity of the peptide.

In the first experiment Etanercept was immobilized using amine coupling on the surface of an CM5 chip. After coupling TNFα was injected at 50 nM (contact time was 1 min at 30 µl/min followed by 1 min dissociation) by itself or with a 2000 times molar excess of RESCA-Z_0185_ or Etanercept to measure the level of inhibition.

In the second experiment TNFα WAS immobilized using amine coupling on the surface of a CM5 chip with the aim to determine the affinity of RESCA-Z_0185_ for TNFα through a single-cycle run. Four concentrations of affibody (1, 5, 25, 125 nM or 50, 100, 250, 500 nM) were subsequently injected during 120 s contact times at 30 µl/min followed by a 300 s dissociation phase. The chip surface was regenerated by 30 s 30 µl/min injections of 10 mM HCl. The data was processed using the Biacore T200 Evaluation Software 3.2.1 (Cytiva).

**SUPPLEMENTARY FIGURES**

**
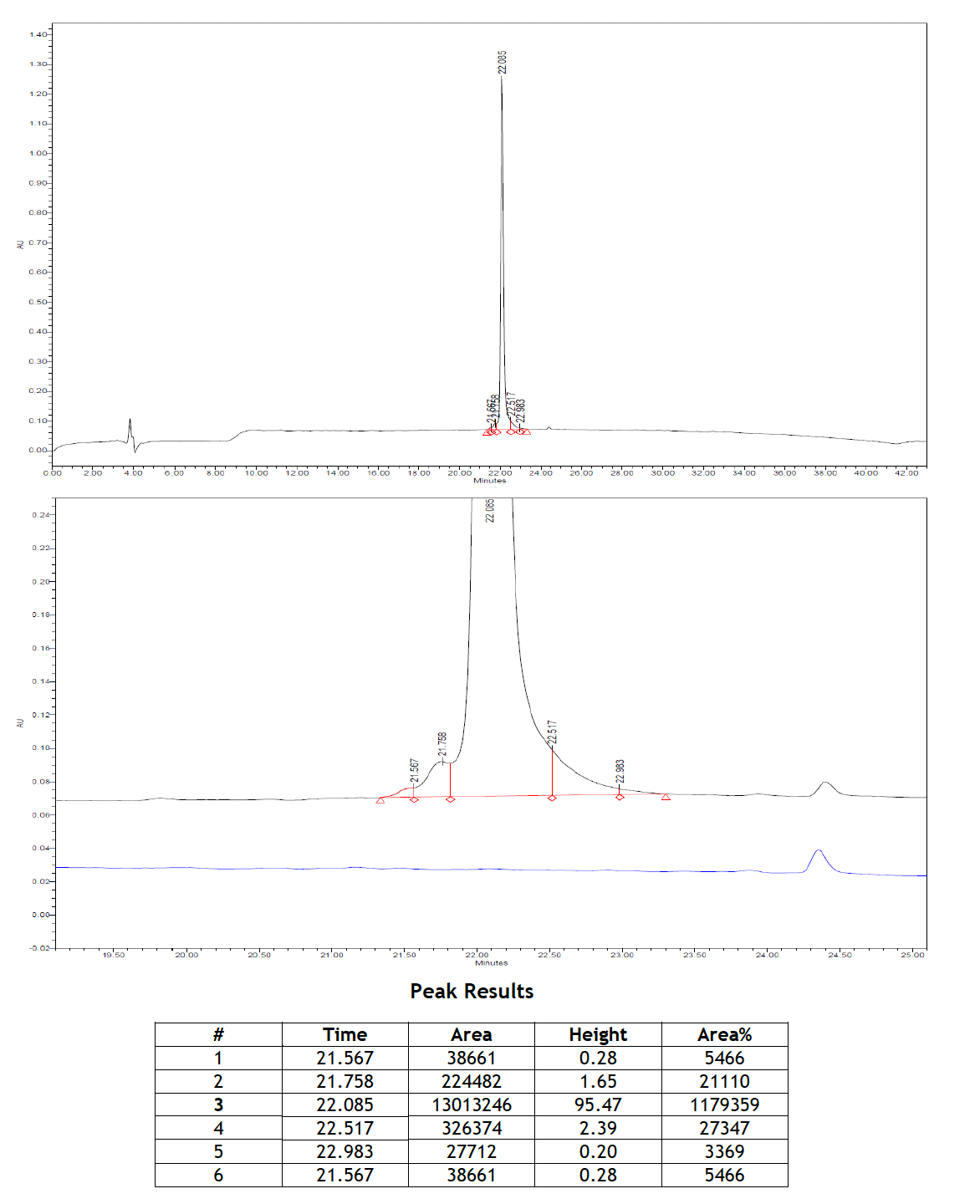
**

**Supplementary Figure 1.** HPLC analysis of RESCA-Z_09591_ (black line) and overlaid blank (blue line) (Luna C18, 250x4.6mm, 2-90%B / 34 minutes, A=0.1% TFA in water, B=0.1% TFA in acetonitrile, 214nm).

**
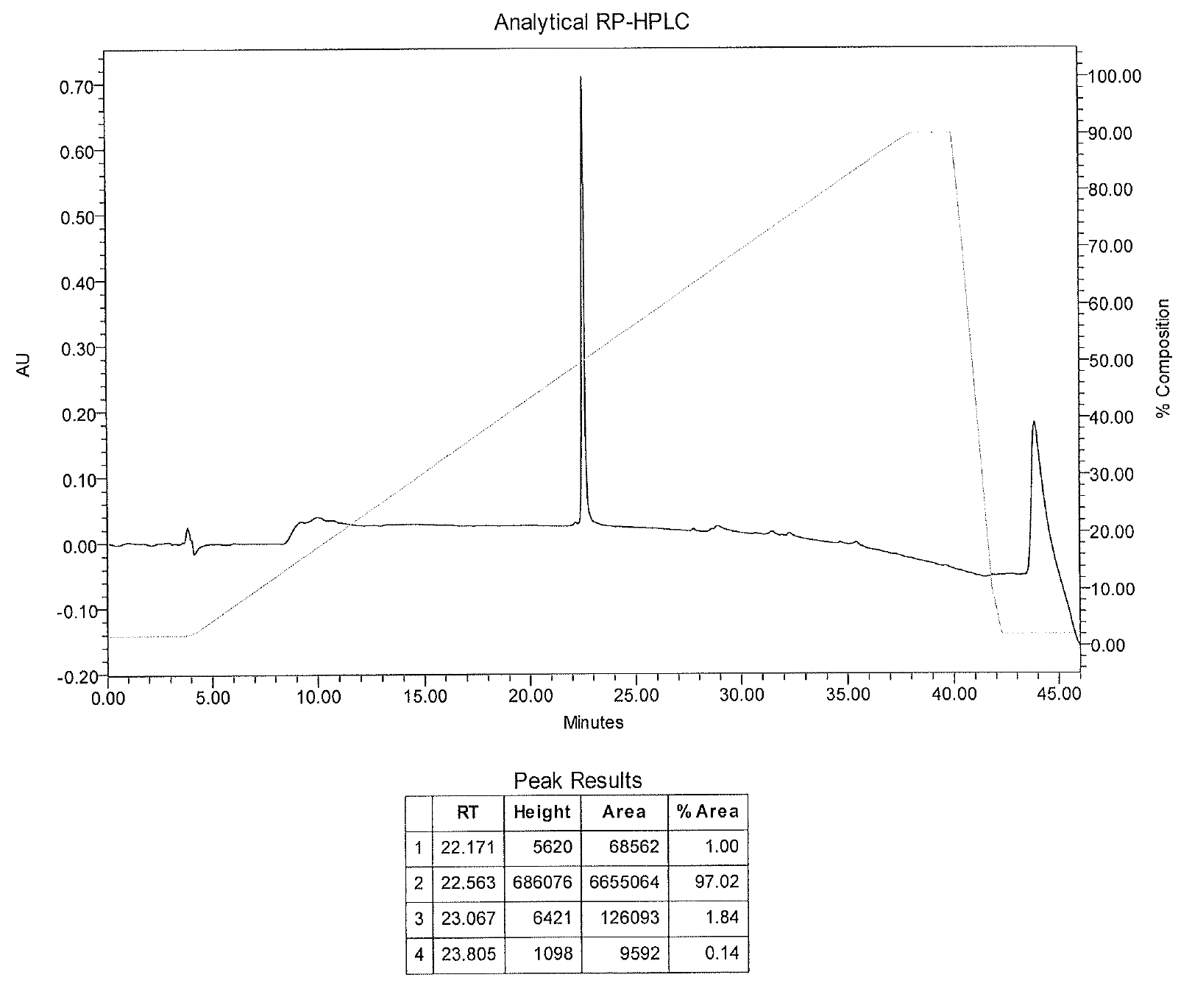
**

**Supplementary Figure 2.** HPLC analysis of RESCA-Z_0185_.

**
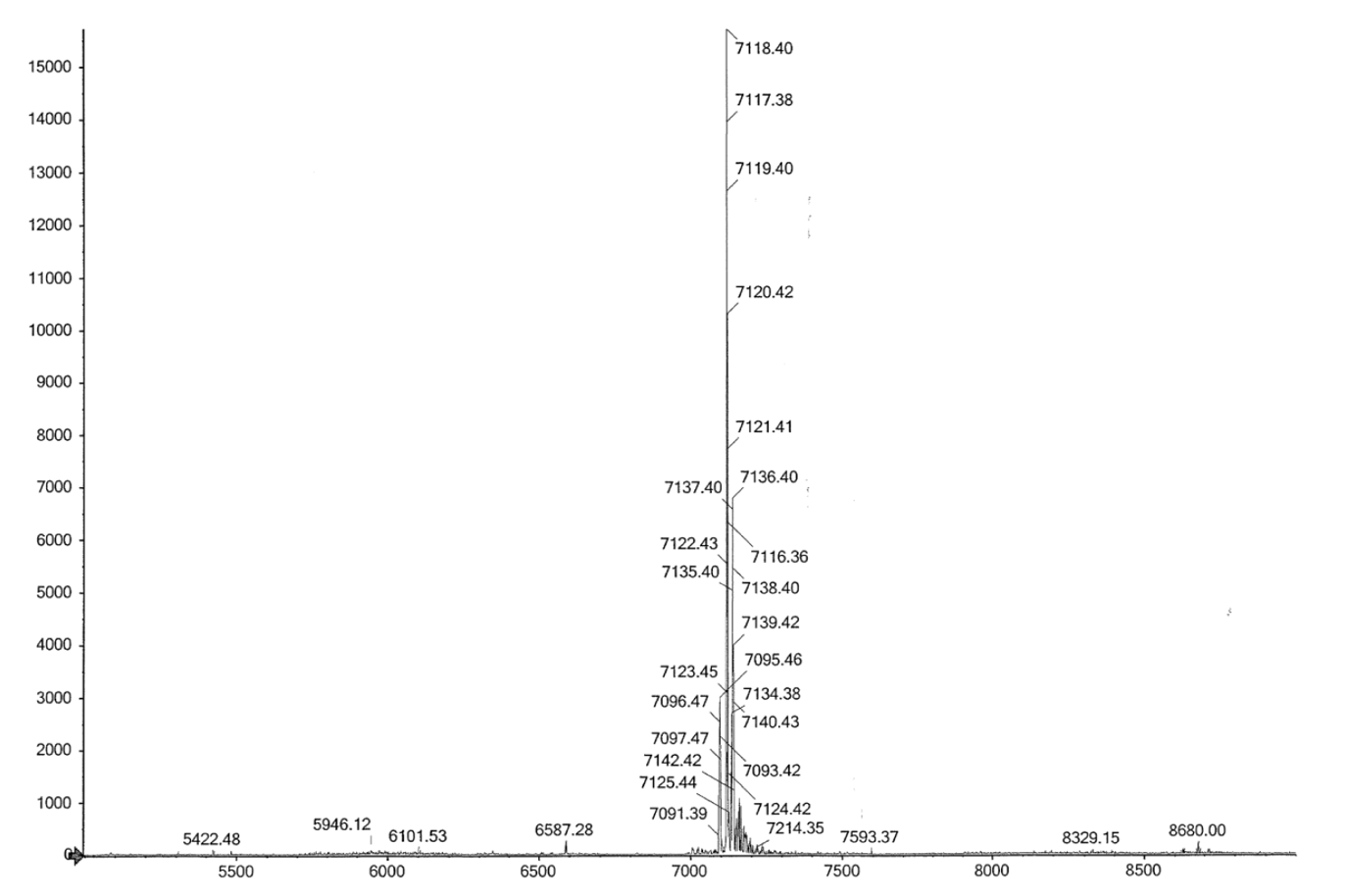
**

**Supplementary Figure 3.** Mass Spectrometry analysis of RESCA-Z_09591_ (MNa+, ESI+)

**
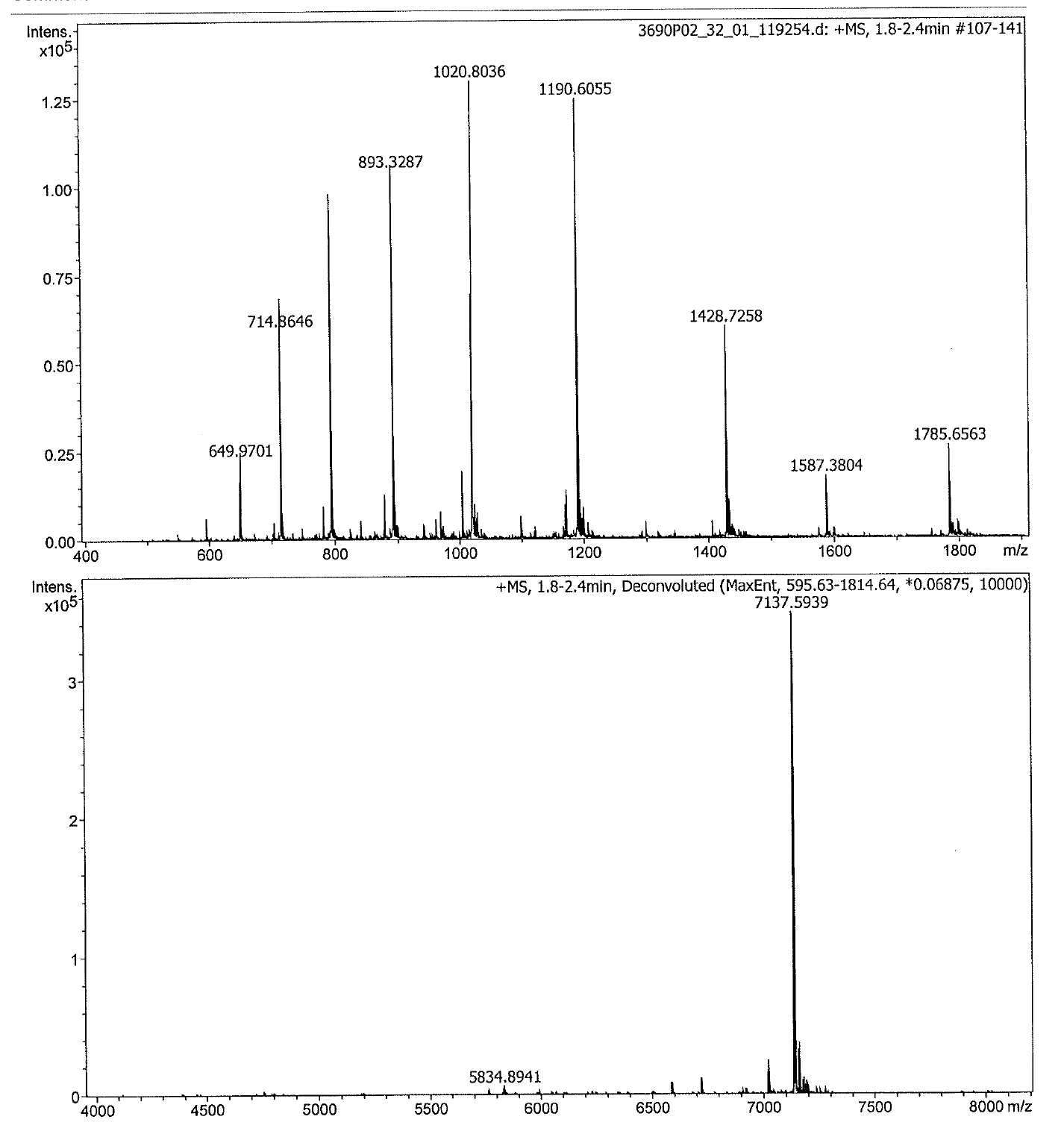
**

**Supplementary Figure 4.** Mass Spectrometry analysis of RESCA-Z_0185_.

**Supplementary Figure 5. Histological staining of the mouse tissue sections examined by Al[^18^F]F-RESCA-Z_09591_.** Liver from mice treated with CCl_4_ developed fibrotic lesions as demonstrated by overlapping staining for collagen (Sirius Red and Massons Trichrome) (Top panels). Liver from healthy liver, on the other hand, were negative for collagen deposition (Tottom panels). Fibrotic liver lesions were positive for PDGFRß, as was the spleen (Top and bottom right panel).

**Supplementary Figure 6. In vitro stability of Al[^18^F]F-RESCA-Z_09591_.** Al[^18^F]F-RESCA-Z_09591_ did not show any degradation for up to two hours in either rat or human plasma (A). representative HPLC chromatograms for the 120 minute time point in rat plasma (B) and human plasma (C).

**Supplementary Figure 7. In vitro binding of Al[^18^F]F-RESCA-Z_0185_ to TNF.** (A) RESCA-Z_0185_ retained an affinity in the single nanomolar range towards human TNF based on two single cycle SPR multi-concentration affinity experiments (5.3±6.3 nM, n=2, representative experiment shown). (B) RESCA-Z_0185_ interfered only weakly with the binding between TNF and Etanercept according to SPR. (C) Radiolabeled Al[^18^F]F-RESCA-Z_0185_ bound to TNF covered wells in an ELISA style assay, with minimal off-target and background binding. Co-incubation partly interfered with the binding to TNF. A star (*) indicates p<0.05 as assessed by one way ANOVA).
